# In search of functional association from time-series microarray data based on the change trend and level of gene expression

**DOI:** 10.1186/1471-2105-7-69

**Published:** 2006-02-15

**Authors:** Feng He, An-Ping Zeng

**Affiliations:** 1 Research Group Systems Biology, GBF – German Research Center for Biotechnology, Mascheroder Weg 1, 38124 Braunschweig, Germany

## Abstract

**Background:**

The increasing availability of time-series expression data opens up new possibilities to study functional linkages of genes. Present methods used to infer functional linkages between genes from expression data are mainly based on a point-to-point comparison. Change trends between consecutive time points in time-series data have been so far not well explored.

**Results:**

In this work we present a new method based on extracting main features of the change trend and level of gene expression between consecutive time points. The method, termed as trend correlation (TC), includes two major steps: 1, calculating a maximal local alignment of change trend score by dynamic programming and a change trend correlation coefficient between the maximal matched change levels of each gene pair; 2, inferring relationships of gene pairs based on two statistical extraction procedures. The new method considers time shifts and inverted relationships in a similar way as the local clustering (LC) method but the latter is merely based on a point-to-point comparison. The TC method is demonstrated with data from yeast cell cycle and compared with the LC method and the widely used Pearson correlation coefficient (PCC) based clustering method. The biological significance of the gene pairs is examined with several large-scale yeast databases. Although the TC method predicts an overall lower number of gene pairs than the other two methods at a same p-value threshold, the additional number of gene pairs inferred by the TC method is considerable: e.g. 20.5% compared with the LC method and 49.6% with the PCC method for a p-value threshold of 2.7E-3. Moreover, the percentage of the inferred gene pairs consistent with databases by our method is generally higher than the LC method and similar to the PCC method. A significant number of the gene pairs only inferred by the TC method are process-identity or function-similarity pairs or have well-documented biological interactions, including 443 known protein interactions and some known cell cycle related regulatory interactions. It should be emphasized that the overlapping of gene pairs detected by the three methods is normally not very high, indicating a necessity of combining the different methods in search of functional association of genes from time-series data. For a p-value threshold of 1E-5 the percentage of process-identity and function-similarity gene pairs among the shared part of the three methods reaches 60.2% and 55.6% respectively, building a good basis for further experimental and functional study. Furthermore, the combined use of methods is important to infer more complete regulatory circuits and network as exemplified in this study.

**Conclusion:**

The TC method can significantly augment the current major methods to infer functional linkages and biological network and is well suitable for exploring temporal relationships of gene expression in time-series data.

## Background

Gene expression profiling has gained a tremendous importance in functional genomic research. The presently often used approach is to compare gene expression in discrete time points resulting for example of different genotypes or cell lines, morbid and healthy (control) objects or under different physiological conditions. This type of static gene expression profiling can already give useful information on the patterns of significantly differentiated expression of genes. However, in order to achieve a more complete picture of significantly differentiated gene expression, especially in order to capture and understand the dynamics of the altered gene expression, it is desirable to measure time-series gene expression [1].

Large efforts have been made in recent years to develop bioinformatics methods to study gene expression patterns [2-5] and/or to infer functional linkages and even regulatory network from microarray data [6-11]. For inference of functional associations among genes two major classes of methods are presently in use. One class of the methods is based on graphical modeling, which include Bayesian network [7] and Gaussian graphical model [12]. Recently, dynamic Bayesian networks have been proposed to model temporal gene expression and represent a promising direction. However, most of the current work in this area is limited to the analysis of a relatively small set of genes due to computational complexity [9-11]. Another class of methods infers functional association from large-scale gene expression data by defining a statistic threshold for the association. The main measure used for defining association is the Pearson correlation coefficient (PCC) [6,13,14]. PCC is widely used for detecting co-expressed genes from both static and time-series expression data. However, several important issues are not specifically addressed in PCC based methods when applied to time-series expression data. A major issue is that the PCC clustering method treats its input as a vector of independent samples and hence doesn't take into account the temporal relationship between consecutive time points. In addition, time-shifted and/or inverted expression of certain gene pairs are not considered. Time-shifted and inverted relationships are important features of gene expression regulation [8]. For example, a gene may activate or inhibit another gene or even several related genes downstream in a regulatory pathway, resulting in time-delayed positive or negative response in the transcription of the downstream gene(s). To consider these phenomena, Qian et al. [8] proposed a local clustering (LC) method. As demonstrated with the expression profiling data of yeast cell cycle this method can identify new, biologically relevant interactions that could not be found by the conventional PCC clustering method. However, the method of Qian et al. [8] is principally still based on a point-to-point comparison or local clustering of expression levels of genes although it explicitly considers time-shifted and inverted gene expression profile. Kwon et al. [15] proposed an 'event-based' edge detection method to consider the change trend of gene expression between consecutive time points. By simplifying a profile of time series into a sequence of decrease or increase events this method is more robust to noises. However, it does not fully make use of the information contained in the gene expression levels in the original data. Filkov et al. [16] proposed a similar method called 'edge detection'. Recently Balasubramaniyan et al. [17] proposed a method to use Spearman rank correlation based on the rank of expressional values. The rank of expressional values is more insensitive to noses or outliers but this method is still based on the point-to-point comparison *per se*.

To more comprehensively consider the temporal relationships of gene expression in time-series microarray data we propose here a new method that is based on extracting the main features of the change trend and the change level of gene expression between consecutive time points. We not only consider the qualitative information (i.e. the change trend) but also the quantitative information (the change level) in the original data. We seek to make the methods more noise-tolerant and at the same time to keep more useful information in the expression values. This new method, termed here as trend correlation (TC), is demonstrated with the microarray data from cell cycle of yeast [18]. The biological significance of functional associations of inferred gene pairs is examined with several large-scale yeast databases. We also extensively compare our method with the LC method and the PCC based clustering method of Eisen et al. [6]. It is shown that a significant number of functionally associated gene pairs, which have well-documented biological interactions and relationships but cannot be significantly detected by the LC and PCC methods, can be inferred by the new method with high statistic significance. The biological significance of the functional association pairs inferred by our method is generally higher than that of the LC method and similar to that of the PCC clustering method which detects however only simultaneous co-expression gene pairs. Furthermore, it is shown that the overlapping of gene pairs detected by the three methods is normally not very high, indicating a necessity of combining the different methods in search of functional association of genes from time-series microarray data.

## Principle and scheme of the proposed method

**Figure 1:**
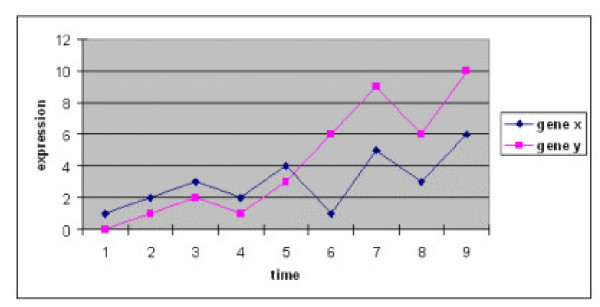
Illustration of calculating the change trend score sc and the correlation coefficient cc of the match change trend. The two genes X and Y in this example have a positive relationship (here expression levels are not normalized). Only the change trend between time points 5 and 6 is different. The number of matched change after time shifts (by dynamic programming) is less than 7. Thus, the maximal match change trend score sc is 7 in this example with 9 time points. To calculate the correlation coefficient, we first extract the change levels of the matched change trends: Xmc12=1,Xmc23=1,Xmc34=−1,Xmc45=2,Xmc56=4,Xmc67=−2,Xmc78=3Ymc12=1,Ymc23=1,Ymc34=−1,Ymc45=2,Ymc56=3,Ymc67=−3,Ymc78=4
 MathType@MTEF@5@5@+=feaafiart1ev1aaatCvAUfKttLearuWrP9MDH5MBPbIqV92AaeXatLxBI9gBaebbnrfifHhDYfgasaacH8akY=wiFfYdH8Gipec8Eeeu0xXdbba9frFj0=OqFfea0dXdd9vqai=hGuQ8kuc9pgc9s8qqaq=dirpe0xb9q8qiLsFr0=vr0=vr0dc8meaabaqaciaacaGaaeqabaqabeGadaaakqaabeqaaiabdIfayjabd2gaTjabdogaJnaaDaaaleaacqaIXaqmaeaacqaIYaGmaaGccqGH9aqpcqaIXaqmcqGGSaalcqWGybawcqWGTbqBcqWGJbWydaqhaaWcbaGaeGOmaidabaGaeG4mamdaaOGaeyypa0JaeGymaeJaeiilaWIaemiwaGLaemyBa0Maem4yam2aa0baaSqaaiabiodaZaqaaiabisda0aaakiabg2da9iabgkHiTiabigdaXiabcYcaSiabdIfayjabd2gaTjabdogaJnaaDaaaleaacqaI0aanaeaacqaI1aqnaaGccqGH9aqpcqaIYaGmcqGGSaalcqWGybawcqWGTbqBcqWGJbWydaqhaaWcbaGaeGynaudabaGaeGOnaydaaOGaeyypa0JaeGinaqJaeiilaWIaemiwaGLaemyBa0Maem4yam2aa0baaSqaaiabiAda2aqaaiabiEda3aaakiabg2da9iabgkHiTiabikdaYiabcYcaSiabdIfayjabd2gaTjabdogaJnaaDaaaleaacqaI3aWnaeaacqaI4aaoaaGccqGH9aqpcqaIZaWmaeaacqWGzbqwcqWGTbqBcqWGJbWydaqhaaWcbaGaeGymaedabaGaeGOmaidaaOGaeyypa0JaeGymaeJaeiilaWIaemywaKLaemyBa0Maem4yam2aa0baaSqaaiabikdaYaqaaiabiodaZaaakiabg2da9iabigdaXiabcYcaSiabdMfazjabd2gaTjabdogaJnaaDaaaleaacqaIZaWmaeaacqaI0aanaaGccqGH9aqpcqGHsislcqaIXaqmcqGGSaalcqWGzbqwcqWGTbqBcqWGJbWydaqhaaWcbaGaeGinaqdabaGaeGynaudaaOGaeyypa0JaeGOmaiJaeiilaWIaemywaKLaemyBa0Maem4yam2aa0baaSqaaiabiwda1aqaaiabiAda2aaakiabg2da9iabiodaZiabcYcaSiabdMfazjabd2gaTjabdogaJnaaDaaaleaacqaI2aGnaeaacqaI3aWnaaGccqGH9aqpcqGHsislcqaIZaWmcqGGSaalcqWGzbqwcqWGTbqBcqWGJbWydaqhaaWcbaGaeG4naCdabaGaeGioaGdaaOGaeyypa0JaeGinaqdaaaa@AB67@ The change level between time points 5 and 6 is excluded because the change (inverted) trend is different from the main (positive) change trend. Using the above matched change levels we can obtain a correlation coefficient cc = 0.9597 according to Eq.1.

The principle of our method is to use information in the change trend and the change level of gene expression between consecutive time points for the inference of functional linkages among genes. To consider not only the positive correlation but also time-shifted and/or inverted expression of certain gene pairs we employ a similar algorithm as the local sequence alignment [19] to calculate a maximal local alignment of change trend (sc) between each gene pair (Fig. 1). We noticed that a significant number of gene pairs that have a same sc value may in fact have a large difference in the change levels of their expression. The distinction of their change levels may lead to the different degrees of similarity of functional association. To solve this problem we make use of the quantitative information in the change levels between consecutive time points and calculate a correlation coefficient (cc) between the maximal alignment.

The method consists of following major steps:

1. Generating a random dataset by shuffling the normalized expression levels at different time points among each gene expression profile in the original dataset;

2. Calculating a maximal local alignment of change trend (sc) between each gene pair in the random dataset as illustrated in Fig. 1 for a simple case;

3. Calculating a correlation coefficient (cc) between the maximal alignment for each gene pair in the random dataset (Fig. 1);

4. Tabulating the frequency of sc (i.e. f(sc)) as function of sc as shown in Fig. S1A [see Additional file 1]; followed by tabulating the distribution of cc for gene pairs which have the same sc;

5. Calculating the conventional p-values for the two scores sc and cc (*P*_*sc*_(*s *>= *sc*), *P*_*cc*_(*c *>= *cc*)) through integration of the frequency distributions (Fig. S1B);

6. Calculating sc and cc between each gene pair in the original dataset;

7. Extraction of functional linkages using Procedure I proposed: extract gene pairs with significantly high sc values with a certain preset p-value. The correlation coefficient cc is regarded as a second index when the gene pairs have the same score sc;

8. Extraction of functional linkages using Procedure II: extract gene pairs with statistically significantly high value of combined scores of sc and cc.

The key steps mentioned above are described in the section of Methods in more detail.  The main programs TC_linkage_infer created in this work can be freely downloaded from our website   http://www.gbf.de/SystemsBiology.

## Results

### Dataset of yeast cell-cycle

We tested our algorithm with the time series microarray data (17 time points) generated by Cho et al. [18] for yeast cell cycle with a whole genome yeast oligonucleotide chip which included over 6000 ORFs. After removing all the negative expression levels in the scaled measurements and all the dubious and genes now deleted in the SGD database [20], 5680 genes were included in our calculation. We examined all the possible pairs among them. The values of the two scores sc and cc and the type of possible relationship (simultaneous, time-shift or inverted) were calculated and assigned for each gene pair.

### Functional associations inferred by different methods

**Figure 2:**
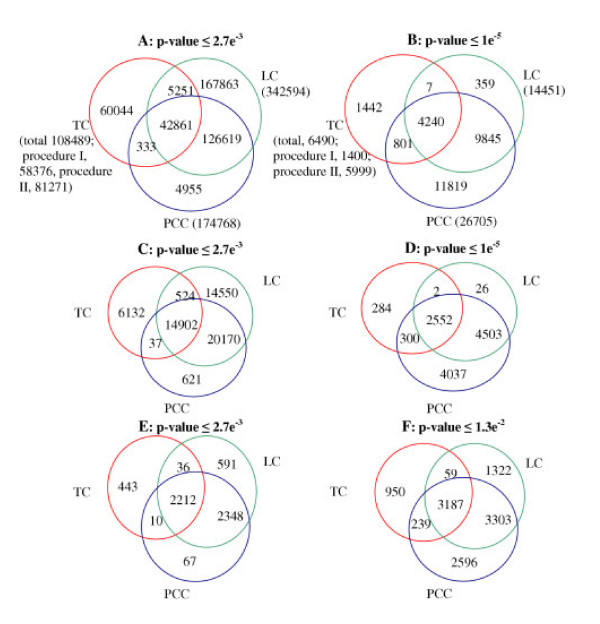
A and B, number of gene pairs detected by the TC method in comparison to those of the LC method and the PCC method inferred from the yeast cell cycle dataset of Cho et al. [18] at two different p-value thresholds. The numbers in parentheses are the whole number of gene pairs detected by the corresponding method. The percentage of the additional number of gene pairs inferred only by the TC method in the text is calculated in a way exemplified in the following by a comparison with those resulted merely from the PCC method at the p-value threshold of 2.7e−3:60044+52514955+126619=49.6%
 MathType@MTEF@5@5@+=feaafiart1ev1aaatCvAUfKttLearuWrP9MDH5MBPbIqV92AaeXatLxBI9gBaebbnrfifHhDYfgasaacH8akY=wiFfYdH8Gipec8Eeeu0xXdbba9frFj0=OqFfea0dXdd9vqai=hGuQ8kuc9pgc9s8qqaq=dirpe0xb9q8qiLsFr0=vr0=vr0dc8meaabaqaciaacaGaaeqabaqabeGadaaakeaacqaIYaGmcqGGUaGlcqaI3aWncqqGLbqzdaahaaWcbeqaaiabgkHiTiabiodaZaaakiabcQda6maalaaabaGaeGOnayJaeGimaaJaeGimaaJaeGinaqJaeGinaqJaey4kaSIaeGynauJaeGOmaiJaeGynauJaeGymaedabaGaeGinaqJaeGyoaKJaeGynauJaeGynauJaey4kaSIaeGymaeJaeGOmaiJaeGOnayJaeGOnayJaeGymaeJaeGyoaKdaaiabg2da9iabisda0iabiMda5iabc6caUiabiAda2iabcwcaLaaa@4DA3@. C and D, number of process-identity pairs among the detected pairs from the three methods; E and F, number of known protein interactions (including protein complexes) in collection dataset of protein interactions [21] among the inferred gene pairs from the three methods.

Figs. 2A and 2B show the number of gene pairs with possible functional association inferred from the yeast time-series data by the TC method (including separate results of the two extraction procedures), the PCC clustering method and the LC method at two different cut-off p-values. The number of gene pairs extracted by Procedure II of our method is generally higher than that from Procedure I, especially at the lower p-value. Note that there is some overlapping between these two extraction procedures so that the total number of the gene pairs inferred is lower than the sum of the two procedures. For comparing the TC method with the other two methods the combined results of the two extraction procedures are considered in the following if not otherwise mentioned. P-values of 2.7E-3 and 1E-5 in the TC method are equivalent to Pearson correlated coefficients of 0.76 and 0.89 in the PCC clustering method and scores of 13 [8] and 15.6 in the local clustering method respectively.

As can be clearly seen in Figs. 2A and 2B, the number of inferred gene pairs depends much on the p-value in all the three methods. In general, the TC method infers a significantly lower number of gene pairs compared to the other two methods. 39.8% and 44.3% of the pairs inferred by the TC method are also found by the PCC and LC methods respectively. The common part of the three methods is somewhat lower and accounts only 12.5% for the LC method, 32.5% for the PCC method and 39.5% for the TC method. The number of gene pairs merely inferred by the TC method is considerable compared with those merely from the LC method (20.5%) and the PCC clustering method (49.6%). By decreasing the p-value threshold to 1E-5, the number of inferred functional pairs decreases remarkably, especially for the TC and LC methods (Fig. 2B). However, the shared part of gene pairs predicted by the TC method increases up to 65.4% and 77.7% compared to the LC and PCC methods respectively, indicating an increased reliability of the prediction (see below for biological significance). The number of additional gene pairs merely inferred by the TC method amounts 1442 and is still significant (Fig. 2B).

### Biological significance of the gene pairs inferred

To assess the biological significance of the inferred functional associations, the gene pairs are compared to known biological processes and protein functions, known protein interactions and regulatory interactions in yeast respectively. The results from the three methods are also compared to each other. The results show that the TC method can significantly enhance the LC and PCC methods to infer functional linkages and biological network, and is well suited to explore temporal relationships of gene expression in time-series data as detailed below.

### Biological process and protein function-similarity gene pairs

In order to generally assess the biological significance of the gene pairs inferred, we first use two databases of biological processes and protein functions classification (supplementary Table S1) [see Additional file 1]. The *S. cerevisiae *Genome Database (SGD) mainly utilizes the Gene Ontology (GO) annotations [20]. We use 32 main biological processes (i.e. conjugation). In this work, if two genes in the pair inferred are involved in the same biological process, we consider the gene pair as a process-identity one. 19.9% of the 108489 gene pairs inferred with the TC method at a p-value threshold of 2.7E-3 (Fig. 2A) are found to be process-identity pairs (Fig. 2C). The detailed distribution of the process-identity pairs in each biological process is listed in Table S3. A similar ratio (20.4%) of process homology gene pairs is found for the 174768 gene pairs inferred by the PCC method. Only 14.6% of the 342594 gene pairs inferred by the LC method with the same p-value cutoff are process-identity pairs. If the results inferred by procedure I and procedure II of the TC method are separately considered, 22.9% of the 58376 gene pairs detected by procedure I are process-identity pairs. The percentage (17.8%) of process-identity pairs among the 81271 gene pairs detected by procedure II is slightly lower than that by procedure I but still somewhat higher than that (14.6%) of the LC method. The additional number (Fig. 2C) of process-identity pairs inferred by the TC method is considerable compared to those resulted only from the LC method (17.8%) and the PCC method (32%) respectively.

Among the 6490 gene pairs (Fig. 2B) predicted by the TC method with a p-value threshold of 1E-5 there are 3138 (48.3%) process-identity pairs (Fig. 2D). Separately, only 32.6% of the gene pairs (1400) detected by procedure I are process-identity pairs, in contrast to as high as 50.8% for the gene pairs by procedure II. Considering the results from p-value ≤ 2.7E-3 mentioned above no general conclusion can be drawn with regard to the question which extraction procedure is more relevant. The percentage of the process-identity pairs among the genes pairs resulted from the LC method and the PCC method is 49% and 42% respectively. Thus, the lower p-value threshold can significantly increase the portion of gene pairs involved in the same biological processes in all the three methods. At this low p-value the additional number (584, Fig. 2D) of process-identity pairs merely inferred by the TC method amounts to 12.9% of those only resulted from the LC method. Compared with those resulted by the PCC method this number declines to 286 (3.3%), suggesting that the gene pairs with a higher ranked functional association inferred by the TC method is more similar to those resulted by the PCC method.

With the percentage of process-identity pairs in the range of 14.6–20.4 at p-value ≤ 2.7E-3 the gene pairs inferred by the three methods seem to have a relatively low biological significance. If the common part of the gene pairs inferred by all the three methods is considered (Fig. 2A), the percentage of process-identity pairs (Fig. 2C) increases to 34.8%, resulting in fairly good biological significance for an *in silico *method of biological function inference. The biological significance can be significantly increased by lowering the p-value. At p-value ≤ 1E-5 the percentage of process-identity pairs ranges from 42 to 49% for the three methods. If the common part of the gene pairs inferred by the three methods at this p-value is considered (Fig. 2B), the percentage of process-identity pairs (Fig. 2D) increases to 60.2%, resulting in a satisfactorily high biological significance.

The second source used in this work for assessing biological relevance of the gene pairs is the Munich Information Center for Protein Sequences (MIPS, [22]) functional catalogue database. For protein functional classification, the MIPS database contains up to 6 different levels within the hierarchy (i.e. metabolism in the first level). We use here the second level of MIPS (i.e. respiration) as Qian et al. [8] did. Altogether 158 function classes are used. It should be mentioned that some functional classes merely belong to protein cellular functions of plants and animals. Here if two genes in the pair inferred have the same protein cellular function, we term the pair as a function-similarity pair. The results of comparison among the three methods (Fig. S2) are similar to those of process-identity pairs, and detailed distribution of the function-similarity pairs in each protein cellular function by the trend correlation method is provided in Table S4 [see Additional file 1]. As in the case of function-similarity pairs, if the common part of the inferred gene pairs (Figs. S2A and S2B) from the three methods is considered the percentage of function-similarity pairs (Figs. S2A and S2B) increases to 31.7% and 55.6% at the two p-values respectively, resulting in a good basis for further experimental and functional studies of the gene pairs inferred.

### Comparison of inferred gene pairs with known protein interactions

To further assess the biological significance of the gene pairs inferred and especially for comparing the three methods we examine here the known protein-protein interactions (including protein complexes) in current databases of yeast among the gene pairs inferred from the yeast cell cycle data by the different methods. Four databases and published high quality datasets (Table S2) are chosen for this purpose.

The protein-protein interactions collection of Yu et al [21] integrates datasets from the databases of MIPS [22], the Database of Interacting Proteins (DIP, [23]), the Biomolecular Interaction Network Database (BIND, [24]) and the experimental datasets of yeast two-hybrid [25,26] and high-throughput mass spectrometry measurements [27,28]. Many of the interactions are manually curated beyond the experimentally derived protein-protein interactions in the three databases mentioned above [21].

**Figure 3:**
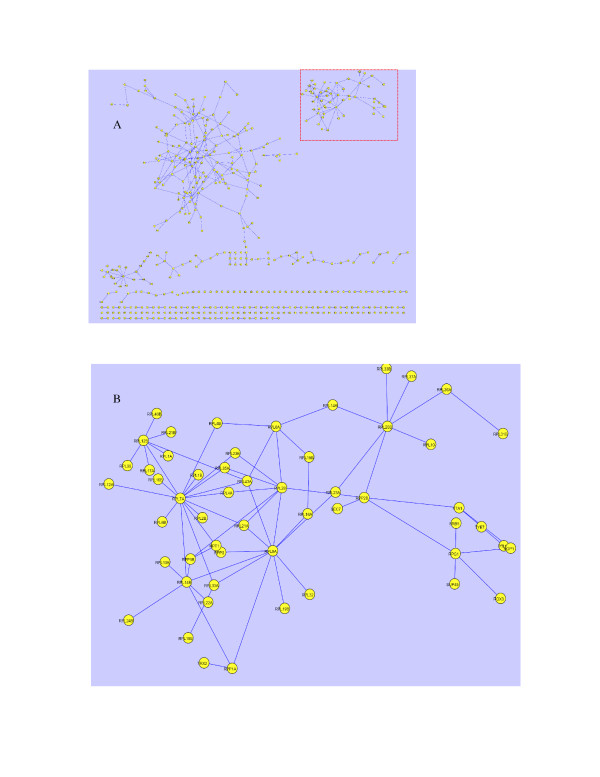
A, Protein interactions (including protein complexes) only detected by the trend correlation method (443 interactions) [see Additional file 2] with a p-value threshold of 2.7E-3 which are known in the protein interactions collection dataset [21]. The network was visualized by Cytoscape   http://www.cytoscape.org/. B, the zooming-in part (red dashed rectangle in the Fig. A) of protein components of the large ribosomal subunit.

With a p-value threshold of 2.7E-3 the TC method detects 2701 gene pairs among the 65160 known protein interactions. Separately, extraction procedure I detects 1152 protein interaction pairs, compared to 2542 pairs by extraction procedure II. With the same p-value threshold 5187 and 4637 such gene pairs can be detected by the LC method and the PCC method respectively. The relatively low number of protein interactions among the gene pairs inferred by the TC method is a consequence of the overall lower number of gene pairs inferred by the TC method (Fig. 2A). In fact, the percentage of protein interaction pairs (Fig. 2E) in the overall gene pairs (Fig. 2A) inferred by the TC method (2701/108489 = 2.49%) is comparable to that of the PCC method (2.65%) but higher than that of LC method (1.51%). Especially the extraction procedure II of the TC method achieves a relatively higher percentage (2542/81271 = 3.13%). It should be mentioned that the low percentage in all these cases is not surprising since only data from cell cycle is used in the inference and the cell cycle represents only a part of cellular functions involving protein-protein interactions in yeast. The results presented in this section are thus more suitable for comparing the different methods rather than for quantitatively assessing the biological significance of the gene pairs inferred. Fig. 2E presents a comparison of the numbers of unique and common pairs of protein-protein interactions inferred by the three methods. The additional pairs (453–479) merely (Fig. 3A) detected by the TC method are significant compared with those resulted merely from the LC method (15.4%) and the PCC method (19.8%). Remarkably, 58 protein-protein interactions among 42 (Fig. 3B) protein components of the large ribosomal subunit of yeast are only significantly detected by the TC method. The fact that the 42 proteins connect with each other by the detected interactions is consistent with the assembling phenomena of components of the large ribosomal subunit. Similarly, 22 interactions among 22 protein components of the small ribosomal subunit of yeast are also only significantly detected by the TC method. The 22 proteins also connect with each other by the 22 interactions (Fig. 3A). Therefore, to have a more complete coverage of the protein interactions it is obvious that the TC method should be combined with the LC and PCC methods.

By loosing the p-value threshold from 2.7E-3 to 1.3E-2 the TC method can detect significantly more pairs (4435) of known protein-protein interactions (Fig. 2F). A p-value of 1.3E-2 in the TC method is equivalent to a Pearson correlated coefficient of 0.6 in the PCC method and a score of 12 in the LC method [29]. With this p-value threshold, 7871 and 9325 pairs can be detected by the LC method and the PCC respectively. The number of protein-protein interactions additionally inferred by the TC method is also considerable (Table 1). When the MIPS, DIP and BIND updated databases are used individually for comparison similar results are obtained and summarized in Table 1. Though the majority of the three databases is consistent some differences exist among them. We chose them to cover more known protein interactions and could therefore obtain more reliable statistics results.

**Table 1:** Results by the trend correlation (TC) method compared to those resulted from the local clustering method (LC) and PCC based clustering method in four protein interactions datasets (p-value threshold 2.7E-3 if not otherwise mentioned).

		Collection dataset	Collection Dataset with p-value ≤ 1.3E-2	MIPS	DIP	BIND
Compared to the LC method	Only TC	453	1189	62	58	111
	Both	2248	3246	84	64	91
	Only LC	2939	4625	393	362	555
	Additional by TC	15.4%	25.7%	15.8%	16.0%	20.0%
Compared to the PCC method	Only TC	479	1009	66	60	112
	Both	2222	3426	80	62	90
	Only PCC	2415	5899	228	243	373
	Additional by TC	19.8%	17.1%	28.9%	24.7%	30.0%

### Comparison with known regulatory interactions

To examine the biological significance of functional associations inferred by the different methods it is also interesting to know if the gene pairs inferred cover some of the known regulatory interactions, especially those involved in the regulation of cell cycle. For this purpose, we use two regulatory datasets (Table S2) as a comparison basis. We first use the dataset including regulatory interactions which are confirmed with a p-value threshold of 1e^-3 ^by genome wide location analysis (GWLA) [30]. With a p-value threshold of 2.7E-3, only a relative low number of the regulatory interactions is detected by the LC method (127), the PCC method (47) and the TC (24) (Fig. S3A). Regulatory interactions detected by the TC method are significantly less than those by the other two methods. Nevertheless, the additional number of regulatory interactions detected by the TC method is considerable compared with those only resulted from the LC method (about 12%) and the PCC method (about 37.8%).

Similar results are obtained when the regulatory interaction collection dataset of Luscombe et al. [31] is used (Fig. S3B). The additional number of interactions predicted by the TC method is 21 (11%) and 22 (about 32%) compared with those resulted only from the LC method and the PCC with a p-value threshold of 2.7E-3. With a p-value threshold of 1.3E-2, the results of comparison are similar to those at a p-value cutoff of 2.7E-3. Some of the typical interactions between transcriptional regulators and target genes which are detected by the TC method but cannot be significantly detected by the LC and/or the PCC method are summarized in Table S5.

A detailed investigation of the results from a p-value threshold of 2.7E-3 shows that the transcriptional factors and/or the targeted genes of 29 [see Additional file 3] among 34 known regulatory interactions inferred by the TC method are involved in cell cycle regulation according to Luscombe et al. [31]. Among the 29 interactions only six interactions have both the activated regulator and the differentially expressed targeted gene according to the gene set of cell cycle regulation given in Luscombe et al. [31]. The fact that 23 of the known interactions have either an inactivated regulator or a non-differentially expressed targeted gene in the cell cycle condition as detected by the TC method with a high p-value cutoff seems to indicate controversies in the designation of activated regulators and differentially expressed genes in the cell cycle condition [31]. For example, the gene FKH2 is annotated in Hollenhorst et al. [32] as a transcriptional factor of the forkhead family that regulates the cell cycle according to SGD. Furthermore, many interactions known to involve FKH2 are detected by at least one of the three methods. However, FKH2 is not regarded as a regulator in the cell cycle condition by Luscombe et al. [31].

**Figure 4:**
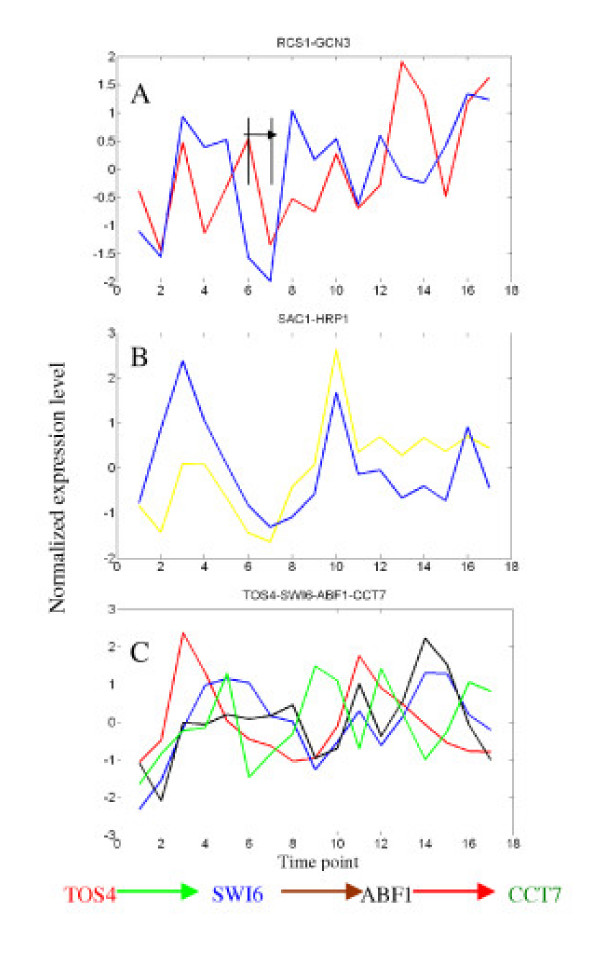
A and B, examples of functional linkages detected only by the new method with a p-value cutoff of 2.7E-3. C, an example of more complete regulatory motifs detected by combining the three methods. Red, regulator; yellow, SAC1 in Fig.B; the legend of linkages in Fig.C is same to that of Fig.5.

We further present two examples of predicted functional linkages which have well-documented biological relationships but cannot be significantly detected by the PCC method and the LC method. The regulatory relationship between RCS1 and GCN3 (Fig. 4A) was confirmed by GWLA with a p-value of 9.2e^-6^. The Pearson correlation coefficient for this gene pair is as low as 0.40 and the score of the LC is only 6.88 (an example of detailed calculation is provided in Table S6). We detect this gene pair with a maximal matched inverted change trend sc of 15 after RCS1 is shifted forward by one time point and obtain a cc value of 0.70 between the matched change levels by the TC method. Another example (Fig. 4B) is the proteins of the genes SAC1 and HRP1 that form a protein complex [21]. Though the score of the LC method is only 10.35 and the Pearson correlation coefficient is 0.44, we could detect this gene pair with a maximal matched similar change trend sc of 15 and obtained a cc value of 0.85 by the TC method.

### The inference of regulatory circuit and network

**Figure 5:**
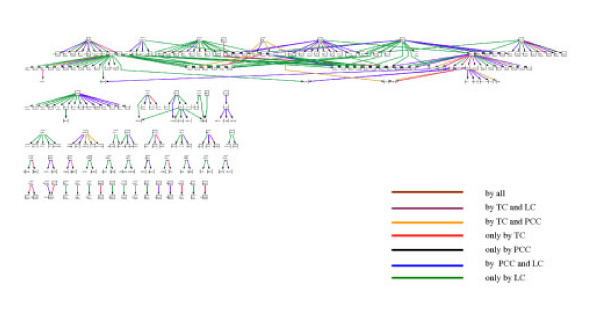
Regulatory network of yeast resulted by combining the three methods with a p-value threshold of 2.7E-3 in the dataset of cell cycle. The network was layout by Cytoscape . For the interactions in this network [see Additional file 3].

As shown above, functional linkages inferred by the individual method do not give a complete picture. It is conceivable that this would be especially the case for inferring regulatory circuits and further for reconstructing regulatory network. Fig. 5 shows comparisons of inferred functional relationships by the individual methods and their combination which are mapped into regulatory circuits (motifs) and networks based on the datasets [30,31]. In consistence with the results of detected regulatory interactions (Fig. S3) the LC method is superior to the other two methods but still gives a very much incomplete picture. The TC and PCC methods can augment the LC method significantly. To infer more complete regulatory circuits and network the three methods should be therefore combined. This is exemplified with two relatively simple regulatory circuits. For the regulatory chain (Fig. 4C) from the regulator TOS4 to the target gene CCT7 the negative relationship between TOS4 and SWI6 can only be detected by the LC method with a shift of two-time-points. The interaction between SWI6 and ABF1 can be detected by all the three methods. However, the interaction between ABF1 and CCT7 can only be detected by the TC method. The three interactions can form one regulatory chain among the complex combination of several motifs (Fig. 5). Another example about a single input motif is explained in Fig. S4 [see Additional file 1].

## Discussion

From a practical point of view there are two key general issues in the analysis of gene expression data. First, we ought to infer functional relationships with high statistic significance and as completely as possible. Second, the functional relationships inferred should have a high biological significance. We proposed a new method in this work and evaluated it mainly regarding these two aspects with the microarray data of yeast cell cycle [18]. The new method is also compared with the local clustering method and the Pearson correlation coefficient based clustering method. The number of functional gene pairs inferred depends very much on the p-value threshold in all the three methods (Fig. 2). With the two p-value cutoffs (2.7E-3 and 1E-5) applied the TC method detects general significantly lower number of gene pairs. Nevertheless, a considerable number of gene pairs is only detected by the TC method, ranging from 20.5% of the number merely detected by the LC method to as high as 49.6% of that merely detected by the PCC method at p-value ≤ 2.7E-3. The ratio of additional gene pairs inferred by our method remains similar (22%) at p-value ≤ 1E-5 compared with the LC method but decreases to a relatively lower value (6.7%) compared with the PCC method. This can be explained by the fact that the statistically higher ranked correlations have mostly simultaneous relationships that can be well detected by all the three methods. Since p-value ≤ 2.7E-3 represents a relatively high statistic significance it can be concluded that the new method detects a significantly high portion of additional functional relationships compared to the other two methods. Since the shared part of gene pairs of the three methods at p-value ≤ 2.7E-3 is less than 50% (12.5–39.5% for the individual method pair) it is also obvious that these methods should be combined to have a more complete exploitation of functional associations of genes buried in time-series expression data.

Concerning the biological significance 19.9% and 48.3% of the gene pairs (Figs. 2A and 2B) inferred by our method (at p-value ≤ 2.7E-3 and 1E-5 respectively) are process-identity ones (Figs. 2C and 2D) by comparing with the known biological processes in the *S. cerevisiae *Genome Database (SGD). This is compared with a process-identity ratio in the range of 14.6–49% for the LC method and 20.4–42 % for the PCC method. Similar results are obtained when the MIPS protein functional catalogue database is used to assess the biological significance (Fig. S2). These results suggest that the gene pairs detected by the three methods achieve fairly good and comparable biological significance. As in the case of the total gene pairs the additional gene pairs with biological significance inferred merely by the trend correlation method is considerable compared to the other two methods (Figs. 2 and S2).

We also examined the gene pairs inferred by the three methods with regard to known protein interactions (Figs. 2E and 2F and Table 1) and known regulatory interactions (Fig. S3) in yeast. In general, the percentage of protein interaction pairs in the overall gene pairs is low and comparable for all the methods (2.5% for our method (3.1% for procedure II), 1.5% for the LC method and 2.7% for the PCC method). This can be understood in view of the fact that only cell cycle data under very specific conditions are used here and the cell cycle represents only a small portion of the cellular activities. Furthermore, only protein interaction pairs with significantly changed expression levels of all the involved partner proteins can be theoretically detected. This applies also to the known regulatory interactions. It is shown that most of the detected regulatory interactions are indeed involved in the cell cycle regulation. Nevertheless, the number of protein and regulatory interactions additionally inferred merely by the trend correlation method is significant in all the cases. It is obvious that more molecular interactions can be obtained if more microarray datasets under different conditions are considered [14].

Given the large number of functionally associated gene pairs inferred by the different methods an important question arises as to how we can find gene pairs which have really a high biological significance and thus would be best candidates for further experimental and functional studies. To this end, the shared part of gene pairs inferred by all the three methods is of particular interest, especially at low p-values. It is found for example that the percentage of process and function-similarity gene pairs of the shared pairs at p-value ≤ 1E-5 can be as high as 60.2% and 55.6%, building a very good basis for experimental study. The common part of the TC and LC methods would be also of particular interest for finding time-delayed and/or inverted functional relationships which have received so far less attention. It should be mentioned that all the functional associated pairs predicted have a high probability to be true and can thus serve as hypotheses for further study in view of the high statistic significance criteria applied. We would also like to emphasize that the combined use of the different methods is not only useful for finding more and highly possible potential candidates of functional association but also very important to infer more complete regulatory circuits and network as exemplified in this study (Figs. 4, 5 and 4).

## Conclusion

The major difference between our method and the other current methods is that the trend correlation method is based on the main change trend and comprehensively considers correlation coefficient between the main change trend of two genes, whereas the other methods are mainly based on the correlation of point-to-point expression levels of two genes. Hence the trend correlation method can reveal additional gene pairs with same function or in the same biological process but yet not significantly co-expressed. It therefore also can infer additional protein-protein and regulatory network as demonstrated. As mentioned above, a combined use of different methods is presently necessary for the analysis of time-series microarray data. As clearly demonstrated in this work the proposed new method can significantly augment the currently major methods and is well suitable for exploring temporal relationships of gene expression in time-series data.

## Methods

### Maximal local alignment of expression change trend

The calculation of the maximal local alignment of expression change trend is similar to the algorithm of local sequence alignment [19] and the local clustering method of Qian et al. [8] for gene expression level. Different from the local clustering method that merely compares the expression levels at each time point, the change trends between time points are used in our method. Considering an expression profiling dataset of n time points the expression ratios at the n time points are first normalized in the "z-score" fashion, resulting in an average expression ratio of zero and a standard deviation of 1 for each gene. The normalized expression level at time point i for gene X is denoted as *X*_*i*_. The change level between time points i and i+1 for gene X is denoted as Xcii+1
 MathType@MTEF@5@5@+=feaafiart1ev1aaatCvAUfKttLearuWrP9MDH5MBPbIqV92AaeXatLxBI9gBaebbnrfifHhDYfgasaacH8akY=wiFfYdH8Gipec8Eeeu0xXdbba9frFj0=OqFfea0dXdd9vqai=hGuQ8kuc9pgc9s8qqaq=dirpe0xb9q8qiLsFr0=vr0=vr0dc8meaabaqaciaacaGaaeqabaqabeGadaaakeaacqWGybawcqWGJbWydaqhaaWcbaGaemyAaKgabaGaemyAaKMaey4kaSIaeGymaedaaaaa@33E9@. The change trend for Xcii+1
 MathType@MTEF@5@5@+=feaafiart1ev1aaatCvAUfKttLearuWrP9MDH5MBPbIqV92AaeXatLxBI9gBaebbnrfifHhDYfgasaacH8akY=wiFfYdH8Gipec8Eeeu0xXdbba9frFj0=OqFfea0dXdd9vqai=hGuQ8kuc9pgc9s8qqaq=dirpe0xb9q8qiLsFr0=vr0=vr0dc8meaabaqaciaacaGaaeqabaqabeGadaaakeaacqWGybawcqWGJbWydaqhaaWcbaGaemyAaKgabaGaemyAaKMaey4kaSIaeGymaedaaaaa@33E9@ is denoted as Xctii+1
 MathType@MTEF@5@5@+=feaafiart1ev1aaatCvAUfKttLearuWrP9MDH5MBPbIqV92AaeXatLxBI9gBaebbnrfifHhDYfgasaacH8akY=wiFfYdH8Gipec8Eeeu0xXdbba9frFj0=OqFfea0dXdd9vqai=hGuQ8kuc9pgc9s8qqaq=dirpe0xb9q8qiLsFr0=vr0=vr0dc8meaabaqaciaacaGaaeqabaqabeGadaaakeaacqWGybawcqWGJbWycqWG0baDdaqhaaWcbaGaemyAaKgabaGaemyAaKMaey4kaSIaeGymaedaaaaa@355A@·Xctii+1
 MathType@MTEF@5@5@+=feaafiart1ev1aaatCvAUfKttLearuWrP9MDH5MBPbIqV92AaeXatLxBI9gBaebbnrfifHhDYfgasaacH8akY=wiFfYdH8Gipec8Eeeu0xXdbba9frFj0=OqFfea0dXdd9vqai=hGuQ8kuc9pgc9s8qqaq=dirpe0xb9q8qiLsFr0=vr0=vr0dc8meaabaqaciaacaGaaeqabaqabeGadaaakeaacqWGybawcqWGJbWycqWG0baDdaqhaaWcbaGaemyAaKgabaGaemyAaKMaey4kaSIaeGymaedaaaaa@355A@ can have only one of the three possible values 1, -1 and 0, corresponding to increase, decrease and no change of gene expression between time points i and i+1 respectively. A matrix of possible alignment between the change trends for gene X and gene Y can then be formed. In our algorithm two matrices P and N are calculated in the following way:

if Xcii+1
 MathType@MTEF@5@5@+=feaafiart1ev1aaatCvAUfKttLearuWrP9MDH5MBPbIqV92AaeXatLxBI9gBaebbnrfifHhDYfgasaacH8akY=wiFfYdH8Gipec8Eeeu0xXdbba9frFj0=OqFfea0dXdd9vqai=hGuQ8kuc9pgc9s8qqaq=dirpe0xb9q8qiLsFr0=vr0=vr0dc8meaabaqaciaacaGaaeqabaqabeGadaaakeaacqWGybawcqWGJbWydaqhaaWcbaGaemyAaKgabaGaemyAaKMaey4kaSIaeGymaedaaaaa@33E9@ = Yctjj+1
 MathType@MTEF@5@5@+=feaafiart1ev1aaatCvAUfKttLearuWrP9MDH5MBPbIqV92AaeXatLxBI9gBaebbnrfifHhDYfgasaacH8akY=wiFfYdH8Gipec8Eeeu0xXdbba9frFj0=OqFfea0dXdd9vqai=hGuQ8kuc9pgc9s8qqaq=dirpe0xb9q8qiLsFr0=vr0=vr0dc8meaabaqaciaacaGaaeqabaqabeGadaaakeaacqWGzbqwcqWGJbWycqWG0baDdaqhaaWcbaGaemOAaOgabaGaemOAaOMaey4kaSIaeGymaedaaaaa@3560@, *P*_*i*, *j *_= *P*_*i*-1, *j*-1_+1; else *P*_*i*, *j *_= *P*_*i*-1, *j*-1_

and

if Xcii+1
 MathType@MTEF@5@5@+=feaafiart1ev1aaatCvAUfKttLearuWrP9MDH5MBPbIqV92AaeXatLxBI9gBaebbnrfifHhDYfgasaacH8akY=wiFfYdH8Gipec8Eeeu0xXdbba9frFj0=OqFfea0dXdd9vqai=hGuQ8kuc9pgc9s8qqaq=dirpe0xb9q8qiLsFr0=vr0=vr0dc8meaabaqaciaacaGaaeqabaqabeGadaaakeaacqWGybawcqWGJbWydaqhaaWcbaGaemyAaKgabaGaemyAaKMaey4kaSIaeGymaedaaaaa@33E9@ * Yctjj+1
 MathType@MTEF@5@5@+=feaafiart1ev1aaatCvAUfKttLearuWrP9MDH5MBPbIqV92AaeXatLxBI9gBaebbnrfifHhDYfgasaacH8akY=wiFfYdH8Gipec8Eeeu0xXdbba9frFj0=OqFfea0dXdd9vqai=hGuQ8kuc9pgc9s8qqaq=dirpe0xb9q8qiLsFr0=vr0=vr0dc8meaabaqaciaacaGaaeqabaqabeGadaaakeaacqWGzbqwcqWGJbWycqWG0baDdaqhaaWcbaGaemOAaOgabaGaemOAaOMaey4kaSIaeGymaedaaaaa@3560@ <0, *N*_*i*, *j *_= *N*_*i*-1, *j*-1_+1; else *N*_*i*, *j *_= *N*_*i*-1, *j*-1_

The initial conditions are *P*_0, *j *_= 0 and *P*_*i*, 0 _= 0, and the same initial conditions are also applied to the matrix of N. The purpose of calculating P and N is to find a local segment that has a maximal aggregated score, namely a maximal match of change trend between two expression patterns. This can be accomplished by using standard dynamic programming [19] as in the local clustering method and results in an alignment of lc matched change trend, where lc ≤ n-1 (altogether n-1 changes among n time points).

Finally, an overall maximal value sc is found by comparing the maximums for matrices P and N. The maximal value is the matched change trend score sc for the two expression patterns. A maximal value from the matrix P means a positively correlated expression pattern of the two genes, whereas a maximal value from the matrix N indicates that these two patterns have an inverted relationship. If the maximum is off-diagonal in its corresponding matrix, then the two expression patterns have a time-shifted relationship. When the number of matched change trends between two genes is relatively small, it is possible that several repeated maximal values exist with different number of shifted-time-points. In this case, we choose the maximal matched change trends with the shortest time shift in the algorithm. Normally these gene pairs are not regarded as inferred linkages because of the small matched change trends based on the p-value threshold.

### Correlation coefficient between the maximal matched change trend of two genes

After obtaining the maximal matched change trend, we need to calculate the correlation coefficient of the maximal matched change trend of the gene pair (Fig. 1). It is calculated according to the following algorithm. Assuming that the final matched change trend for gene X and gene Y are Xctxf−1xf
 MathType@MTEF@5@5@+=feaafiart1ev1aaatCvAUfKttLearuWrP9MDH5MBPbIqV92AaeXatLxBI9gBaebbnrfifHhDYfgasaacH8akY=wiFfYdH8Gipec8Eeeu0xXdbba9frFj0=OqFfea0dXdd9vqai=hGuQ8kuc9pgc9s8qqaq=dirpe0xb9q8qiLsFr0=vr0=vr0dc8meaabaqaciaacaGaaeqabaqabeGadaaakeaacqWGybawcqWGJbWycqWG0baDdaqhaaWcbaGaemiEaGNaemOzayMaeyOeI0IaeGymaedabaGaemiEaGNaemOzaygaaaaa@384B@ and Yctyf−1yf
 MathType@MTEF@5@5@+=feaafiart1ev1aaatCvAUfKttLearuWrP9MDH5MBPbIqV92AaeXatLxBI9gBaebbnrfifHhDYfgasaacH8akY=wiFfYdH8Gipec8Eeeu0xXdbba9frFj0=OqFfea0dXdd9vqai=hGuQ8kuc9pgc9s8qqaq=dirpe0xb9q8qiLsFr0=vr0=vr0dc8meaabaqaciaacaGaaeqabaqabeGadaaakeaacqWGzbqwcqWGJbWycqWG0baDdaqhaaWcbaGaemyEaKNaemOzayMaeyOeI0IaeGymaedabaGaemyEaKNaemOzaygaaaaa@3851@ respectively in the maximal alignment, where xf and yf refer to the last time points of genes X and Y in the maximal alignment and we assign xf ≤ yf. We then give a new index for the time points of match trends, where the matched change levels for genes X and Y are now denoted as *Xmc *and *Ymc *in the following algorithm:

Do while 2 ≤ i ≤ xf and yf - xf + 2 ≤ j ≤ yf

If sc from the matrix P and Xcti−1i
 MathType@MTEF@5@5@+=feaafiart1ev1aaatCvAUfKttLearuWrP9MDH5MBPbIqV92AaeXatLxBI9gBaebbnrfifHhDYfgasaacH8akY=wiFfYdH8Gipec8Eeeu0xXdbba9frFj0=OqFfea0dXdd9vqai=hGuQ8kuc9pgc9s8qqaq=dirpe0xb9q8qiLsFr0=vr0=vr0dc8meaabaqaciaacaGaaeqabaqabeGadaaakeaacqWGybawcqWGJbWycqWG0baDdaqhaaWcbaGaemyAaKMaeyOeI0IaeGymaedabaGaemyAaKgaaaaa@3565@ = Yctj−1j
 MathType@MTEF@5@5@+=feaafiart1ev1aaatCvAUfKttLearuWrP9MDH5MBPbIqV92AaeXatLxBI9gBaebbnrfifHhDYfgasaacH8akY=wiFfYdH8Gipec8Eeeu0xXdbba9frFj0=OqFfea0dXdd9vqai=hGuQ8kuc9pgc9s8qqaq=dirpe0xb9q8qiLsFr0=vr0=vr0dc8meaabaqaciaacaGaaeqabaqabeGadaaakeaacqWGzbqwcqWGJbWycqWG0baDdaqhaaWcbaGaemOAaOMaeyOeI0IaeGymaedabaGaemOAaOgaaaaa@356B@ then

Xmcp−1p=Xci−1i
 MathType@MTEF@5@5@+=feaafiart1ev1aaatCvAUfKttLearuWrP9MDH5MBPbIqV92AaeXatLxBI9gBaebbnrfifHhDYfgasaacH8akY=wiFfYdH8Gipec8Eeeu0xXdbba9frFj0=OqFfea0dXdd9vqai=hGuQ8kuc9pgc9s8qqaq=dirpe0xb9q8qiLsFr0=vr0=vr0dc8meaabaqaciaacaGaaeqabaqabeGadaaakeaacqWGybawcqWGTbqBcqWGJbWydaqhaaWcbaGaemiCaaNaeyOeI0IaeGymaedabaGaemiCaahaaOGaeyypa0JaemiwaGLaem4yam2aa0baaSqaaiabdMgaPjabgkHiTiabigdaXaqaaiabdMgaPbaaaaa@3DCB@

Ymcp−1p=Ycj−1j
 MathType@MTEF@5@5@+=feaafiart1ev1aaatCvAUfKttLearuWrP9MDH5MBPbIqV92AaeXatLxBI9gBaebbnrfifHhDYfgasaacH8akY=wiFfYdH8Gipec8Eeeu0xXdbba9frFj0=OqFfea0dXdd9vqai=hGuQ8kuc9pgc9s8qqaq=dirpe0xb9q8qiLsFr0=vr0=vr0dc8meaabaqaciaacaGaaeqabaqabeGadaaakeaacqWGzbqwcqWGTbqBcqWGJbWydaqhaaWcbaGaemiCaaNaeyOeI0IaeGymaedabaGaemiCaahaaOGaeyypa0JaemywaKLaem4yam2aa0baaSqaaiabdQgaQjabgkHiTiabigdaXaqaaiabdQgaQbaaaaa@3DD3@

Endif

If sc from the matrix N and Xcti−1i
 MathType@MTEF@5@5@+=feaafiart1ev1aaatCvAUfKttLearuWrP9MDH5MBPbIqV92AaeXatLxBI9gBaebbnrfifHhDYfgasaacH8akY=wiFfYdH8Gipec8Eeeu0xXdbba9frFj0=OqFfea0dXdd9vqai=hGuQ8kuc9pgc9s8qqaq=dirpe0xb9q8qiLsFr0=vr0=vr0dc8meaabaqaciaacaGaaeqabaqabeGadaaakeaacqWGybawcqWGJbWycqWG0baDdaqhaaWcbaGaemyAaKMaeyOeI0IaeGymaedabaGaemyAaKgaaaaa@3565@ * Yctj−1j
 MathType@MTEF@5@5@+=feaafiart1ev1aaatCvAUfKttLearuWrP9MDH5MBPbIqV92AaeXatLxBI9gBaebbnrfifHhDYfgasaacH8akY=wiFfYdH8Gipec8Eeeu0xXdbba9frFj0=OqFfea0dXdd9vqai=hGuQ8kuc9pgc9s8qqaq=dirpe0xb9q8qiLsFr0=vr0=vr0dc8meaabaqaciaacaGaaeqabaqabeGadaaakeaacqWGzbqwcqWGJbWycqWG0baDdaqhaaWcbaGaemOAaOMaeyOeI0IaeGymaedabaGaemOAaOgaaaaa@356B@ < 0 then

Xmcn−1n=Xci−1i
 MathType@MTEF@5@5@+=feaafiart1ev1aaatCvAUfKttLearuWrP9MDH5MBPbIqV92AaeXatLxBI9gBaebbnrfifHhDYfgasaacH8akY=wiFfYdH8Gipec8Eeeu0xXdbba9frFj0=OqFfea0dXdd9vqai=hGuQ8kuc9pgc9s8qqaq=dirpe0xb9q8qiLsFr0=vr0=vr0dc8meaabaqaciaacaGaaeqabaqabeGadaaakeaacqWGybawcqWGTbqBcqWGJbWydaqhaaWcbaGaemOBa4MaeyOeI0IaeGymaedabaGaemOBa4gaaOGaeyypa0JaemiwaGLaem4yam2aa0baaSqaaiabdMgaPjabgkHiTiabigdaXaqaaiabdMgaPbaaaaa@3DC3@

Ymcn−1n=Ycj−1j
 MathType@MTEF@5@5@+=feaafiart1ev1aaatCvAUfKttLearuWrP9MDH5MBPbIqV92AaeXatLxBI9gBaebbnrfifHhDYfgasaacH8akY=wiFfYdH8Gipec8Eeeu0xXdbba9frFj0=OqFfea0dXdd9vqai=hGuQ8kuc9pgc9s8qqaq=dirpe0xb9q8qiLsFr0=vr0=vr0dc8meaabaqaciaacaGaaeqabaqabeGadaaakeaacqWGzbqwcqWGTbqBcqWGJbWydaqhaaWcbaGaemOBa4MaeyOeI0IaeGymaedabaGaemOBa4gaaOGaeyypa0JaemywaKLaem4yam2aa0baaSqaaiabdQgaQjabgkHiTiabigdaXaqaaiabdQgaQbaaaaa@3DCB@

Endif

Loop

In the following equation k corresponds to p or n in the above loop.

cc(x,y)=|1sc∗∑k=2,sc+1(Xmck−1k−Xmc¯σXmc)(Ymck−1k−Ymc¯σYmc)|     Eq.1
 MathType@MTEF@5@5@+=feaafiart1ev1aaatCvAUfKttLearuWrP9MDH5MBPbIqV92AaeXatLxBI9gBaebbnrfifHhDYfgasaacH8akY=wiFfYdH8Gipec8Eeeu0xXdbba9frFj0=OqFfea0dXdd9vqai=hGuQ8kuc9pgc9s8qqaq=dirpe0xb9q8qiLsFr0=vr0=vr0dc8meaabaqaciaacaGaaeqabaqabeGadaaakeaacqWGJbWycqWGJbWydaWgaaWcbaGaeiikaGIaemiEaGNaeiilaWIaemyEaKNaeiykaKcabeaakiabg2da9maaemaabaWaaSaaaeaacqaIXaqmaeaacqWGZbWCcqWGJbWyaaGaey4fIOYaaabuaeaacqGGOaakdaWcaaqaaiabdIfayjabd2gaTjabdogaJnaaDaaaleaacqWGRbWAcqGHsislcqaIXaqmaeaacqWGRbWAaaGccqGHsisldaqdaaqaaiabdIfayjabd2gaTjabdogaJbaaaeaacqaHdpWCdaWgaaWcbaGaemiwaGLaemyBa0Maem4yamgabeaaaaGccqGGPaqkcqGGOaakdaWcaaqaaiabdMfazjabd2gaTjabdogaJnaaDaaaleaacqWGRbWAcqGHsislcqaIXaqmaeaacqWGRbWAaaGccqGHsisldaqdaaqaaiabdMfazjabd2gaTjabdogaJbaaaeaacqaHdpWCdaWgaaWcbaGaemywaKLaemyBa0Maem4yamgabeaaaaGccqGGPaqkaSqaaiabdUgaRjabg2da9iabikdaYiabcYcaSiabdohaZjabdogaJjabgUcaRiabigdaXaqab0GaeyyeIuoaaOGaay5bSlaawIa7aiaaxMaacaWLjaGaeeyrauKaeeyCaeNaeiOla4IaeGymaedaaa@78FF@

Where

*cc*_(*x*, *y*) _is the correlation coefficient between the maximal matched change trends of genes X and Y. Xmc¯
 MathType@MTEF@5@5@+=feaafiart1ev1aaatCvAUfKttLearuWrP9MDH5MBPbIqV92AaeXatLxBI9gBaebbnrfifHhDYfgasaacH8akY=wiFfYdH8Gipec8Eeeu0xXdbba9frFj0=OqFfea0dXdd9vqai=hGuQ8kuc9pgc9s8qqaq=dirpe0xb9q8qiLsFr0=vr0=vr0dc8meaabaqaciaacaGaaeqabaqabeGadaaakeaadaqdaaqaaiabdIfayjabd2gaTjabdogaJbaaaaa@30A8@ and Ymc¯
 MathType@MTEF@5@5@+=feaafiart1ev1aaatCvAUfKttLearuWrP9MDH5MBPbIqV92AaeXatLxBI9gBaebbnrfifHhDYfgasaacH8akY=wiFfYdH8Gipec8Eeeu0xXdbba9frFj0=OqFfea0dXdd9vqai=hGuQ8kuc9pgc9s8qqaq=dirpe0xb9q8qiLsFr0=vr0=vr0dc8meaabaqaciaacaGaaeqabaqabeGadaaakeaadaqdaaqaaiabdMfazjabd2gaTjabdogaJbaaaaa@30AA@ are the mean of the maximal matched change levels of genes X and Y respectively σ_*Xmc *_and σ_*Ymc *_are the standard deviation of the maximal matched change levels.

### Significance statistics

In order to estimate the p-value for a given score a dataset of random expression patterns was generated by shuffling the normalized expression levels of the original data at different time points. To assure the reliability of p-value we generated the random expression patterns three times, resulting in calculations for about 4.9e^7 ^gene pairs. Using the algorithm described above we calculated the maximal matched change trend scores sc and the correlation coefficient cc between the maximal matched change trend for each gene pair in the random expression dataset. We first tabulated the frequency (f(sc)) of sc and then the distribution of cc for pairs which have the same sc value. Through integration we could calculate the conventional p-value for the two scores sc (Fig. S1B) and cc (Fig. S1C) (*P*_*sc*_(*s *>= *sc*), *P*_*cc*_(*c *>= *cc*)). They are defined as the probability of obtaining a score (s or c) larger than or equivalent to sc or cc from the random patterns. The higher the matched change trend score is, the more likely the gene pair is correlated (positively or negatively). The higher the correlated coefficient, the more likely the gene pairs are correlated for a given value of sc.

### Extraction procedure I

As shown in Fig. S1B the probability to achieve a high sc score in the random dataset is very low. For example, the p-value for gene pairs with sc ≥ 15 is as low as 1.7e^-3 ^and with sc ≥ 16 as low as 1.1e^-4^. Hence, if two genes in the original expression data have a sc score ≥ 15 it is very likely that these two genes have a functional association that would be expected with a low probability by chance. For a given significant p-value threshold such as 2.7E-3, because the p-value of gene pairs with sc ≥ 15 is only 1.7e^-3 ^that is lower than the threshold, all the gene pairs with sc ≥ 15 can be regarded as having functional relationships. But *P*_*sc *_for gene pairs with sc ≥ 14 is about 1.27e^-2 ^that is higher than the threshold 2.7E-3, only parts of the gene pairs with sc = 14 should be extracted. Among the gene pairs with sc = 14 the pairs with a higher cc value (Fig. S1C) should have a higher possibility to have a functional association. Considering these facts and the distribution frequency of sc we propose to define the following overall p-value to better reflect the significance statistics for inferring functional associations among genes:

*P *= *P*_*sc*_(*sc *+ 1) + (*P*_*sc*_(*sc*) - *P*_*sc*_(*sc *+ 1)) * *P*_*cc *_or *P *= *P*_*sc*_(*sc *+ 1) + *f*(*sc*) * *P*_*cc *_

where if sc is the highest possible score (here 16 in the yeast cell cycle dataset with 17 time points), then *P*_*sc*_(*sc *+ 1) = 0. This overall p-value combines the p-values for both sc and cc and the distribution frequency of sc. If the sc and cc values of a gene pair in original expression dataset result in an overall p-value less than a certain threshold, this gene pair is considered as functionally associated. This extraction procedure is called here Procedure I.

### Extraction procedure II

In applying extraction Procedure I we noticed that there are many gene pairs the change trend score (sc) of which is not very high, but the correlation coefficient between the maximal change levels of which is significantly high. These gene pairs will not be extracted according to Procedure I because of the relatively high *P*_*sc *_values at low sc values (Fig. S1B). However, they may in fact also have a high possibility of functional association since the main change levels are correlated well. The difference of some of the change trends from the main trend between the two genes may be caused by the involvement of multi-regulators in some time regions or by measurement errors and expression noises. For these reasons, we propose a second procedure (Procedure II) to extract gene pairs with possible functional relationships. In Procedure II, if the correlation coefficient cc is not considered, the likelihood of functional association of all the pairs increases with increasing sc if sc is higher than or equal to a characteristic score sc_m_. This characteristic score sc_m _is defined as the sc that has the highest frequency in the random dataset (Fig. S1A, here sc_m _10). If sc is smaller than sc_m_, the frequency of pairs with sc reduces along with the decrease of sc, as does also the possibility for a functional association of the gene pair. Because the possibility for functional association should have a reverse relationship with the frequency of the corresponding sc, one should not consider gene pairs with sc smaller than sc_m_. We therefore propose to extract gene pairs through the formulas:

P-value of cc cutoff for each sc: Pcc(i)=ff(sci)(i=scm to scmax⁡)
 MathType@MTEF@5@5@+=feaafiart1ev1aaatCvAUfKttLearuWrP9MDH5MBPbIqV92AaeXatLxBI9gBaebbnrfifHhDYfgasaacH8akY=wiFfYdH8Gipec8Eeeu0xXdbba9frFj0=OqFfea0dXdd9vqai=hGuQ8kuc9pgc9s8qqaq=dirpe0xb9q8qiLsFr0=vr0=vr0dc8meaabaqaciaacaGaaeqabaqabeGadaaakeaacqWGqbaudaWgaaWcbaGaem4yamMaem4yamgabeaakiabcIcaOiabdMgaPjabcMcaPiabg2da9maalaaabaGaemOzaygabaGaemOzayMaeiikaGIaem4CamNaem4yam2aaSbaaSqaaiabdMgaPbqabaGccqGGPaqkaaGaeiikaGIaeeyAaKMaeyypa0Jaee4CamNaee4yam2aaSbaaSqaaiabb2gaTbqabaGccqqGGaaicqqG0baDcqqGVbWBcqqGGaaicqqGZbWCcqqGJbWydaWgaaWcbaGagiyBa0MaeiyyaeMaeiiEaGhabeaakiabcMcaPaaa@5151@

Where f=pm
 MathType@MTEF@5@5@+=feaafiart1ev1aaatCvAUfKttLearuWrP9MDH5MBPbIqV92AaeXatLxBI9gBaebbnrfifHhDYfgasaacH8akY=wiFfYdH8Gipec8Eeeu0xXdbba9frFj0=OqFfea0dXdd9vqai=hGuQ8kuc9pgc9s8qqaq=dirpe0xb9q8qiLsFr0=vr0=vr0dc8meaabaqaciaacaGaaeqabaqabeGadaaakeaacqWGMbGzcqGH9aqpdaWcaaqaaiabdchaWbqaaiabd2gaTbaaaaa@31E3@

sc_m_, sc with the highest frequency.

sc_max_, the maximal possible sc score in the time series dataset

m, the number between sc_m _and sc_max_

p, the given significant p-value threshold

f, the p-value threshold for each sc (between sc_m _and sc_max_)

*f*(*sc*_*i*_), the frequency of gene pairs with sc_i_

If the p-value of cc from a gene pair with sc_i _in the original expression dataset as determined in Fig. S1C is less than the corresponding *P*_*cc*_(*i*), this gene pair is considered as functionally associated in the extraction procedure II.

## Authors' contributions

FH initially proposed the method, wrote the programs and drafted the manuscript. APZ made suggestions to improve the method, supervised the study and revised the manuscript.

## Supplementary Material

Additional File 1 Supplementary material four supplementary figures and six supplementary tables. Click here for file

Additional File 2 Table of protein-protein interactions in Fig. 3 all the protein-protein interactions and comparison among three methods in the figure 3. Click here for file

Additional File 3 Table of regulatory interactions in Fig. 5 all the regulatory interactions in the figure 5 and the corresponding scores by the three methods. Click here for file
