# Supplementary material for: In search of functional association from time-series microarray data based on the change trend and level of gene expression
Source: BMC Bioinformatics. 2006 Feb 15;7:69. doi: 10.1186/1471-2105-7-69 (PMC1435774; doi:10.1186/1471-2105-7-69)
Supplement: Additional File 1 — Supplementary material four supplementary figures and six supplementary tables. [file 1471-2105-7-69-S1.doc]

# Supplementary materials to the manuscript

**In search of functional association from time-series microarray data based on the change trend and level of gene expression**

By Feng He and An-Ping Zeng

2005-06

**List**

**Four supplementary figures**

**Six supplementary tables**

###

**A**

**B**

**C**

Fig.S1. A. Frequency of sc; B: p-value for sc; C: p-value for cc at each sc in the randomly shuffled expression data of yeast cell cycle (Cho et al.[18]. If a gene pair has an sc value of 14 and a cc value of 0.86, an overall p-value is calculated as 2.3e-3 (with $P_{sc}(15)$= 0.0017 and $P_{sc}(14)$= 0.0127 and$P_{cc}$= 0.0573). With a threshold p-value of 2.7e-3, this gene pair is considered to be functionally associated with a statistically high probability in the extraction procedure I proposed.

250

259

3

45

4326

2359

3856

**B: p-value ≤ 1e-5**

TC

LC

PCC

LC

6948

42

598

19918

22186

13589

705

**A: p-value ≤ 2.7e-3**

TC

PCC

Fig.S2. Function-similarity pairs (based on MIPS database) inferred by the TC method versus those resulted from the LC method and the conventional PCC clustering method.

32

1

2

196

87

25

86

**p-value ≤ 1.3e-2**

TC

LC

PCC

LC

(127)

14

0

0

81

36

10

1

**p-value ≤ 2.7e-3**

TC

(24)

PCC (47)

**A**

LC

58

4

6

317

147

19

116

**p-value ≤ 1.3e-2**

TC

PCC

LC

(199)

21

0

1

125

66

8

3

**p-value ≤ 2.7e-3**

TC

(30)

PCC (77)

**B**

.

Fig.S3. A, results by the TC method vs. the LC method and the PCC clustering method respectively according to the dataset of genome wide location analysis (Lee et al. [14]); B, results by the TC method vs. the LC method and the PCC clustering method respectively according to the regulatory interactions collection dataset (Luscombe et al. [31]). The number in parenthesis is the whole number of regulatory interactions detected by the corresponding method with a p-value threshold of 2.7e-3.

**
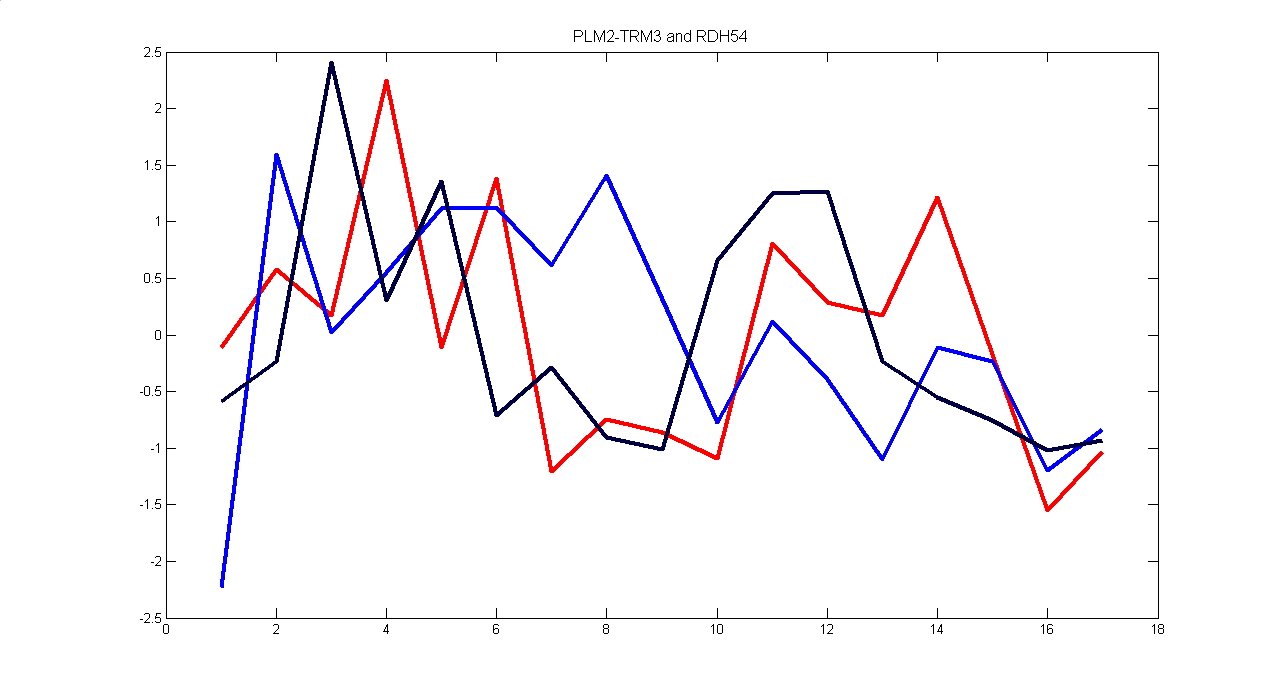
**

PLM2

TRM3

RDH54

Normalized expression level

Time point

Fig.S4. Another example of more complete regulatory motifs detected by combining the three methods. The legend of linkages is same to that of Fig.5 (For details see text). The transcriptional regulator PLM2 is known to regulate TRM3 and RDH54 forming one part of a single input motif (Lee et al., [14]; Luscombe et al., [31]). But the two interactions between the regulator PLM2 and target genes TRM3 and RDH54 can only be significantly detected by TC method and LC method, respectively.

Table S1. Databases of biological processes and protein cellular function classification.

| Database | Number of terms | Downloaded date |
| --- | --- | --- |
| SGD | 32 | 01-20-2005 |
| MIPS | 158 | 02-15-2005 |

Note: In this work, we have chosen all the biological processes in the list of advanced search in SGD except for the class biological process unknown.

Table S2. Databases or datasets of protein-protein interactions and regulatory interactions.

| Type | Datasets | Number of gene pairs | Downloaded date |
| --- | --- | --- | --- |
| Protein interactions | Collection dataset  (Yu et al., [21]) | 65160 | Published  01-14-2004 |
|  | MIPS | 13895 | 01-18-2005 |
|  | DIP | 14187 | 02-06-2005 |
|  | BIND | 27480 | 02-02-2005 |
| Regulatory interactions | Genome wide location analysis  (Lee et al., [14]) | 3760 | Published  10-25-2002 |
|  | Collection dataset (Luscombe et al., [31]) | 6105 | Published  09-16-2004 |

Note: in the six datasets, we exclude the pairs with two same genes and the pairs with genes which don’t exist in the used Cho cell cycle dataset.

Table S3. Distribution of process-identity pairs inferred by the proposed method (with a p-value threshold of 2.7e-3) in each biological process class of the database SGD.

| Biological process | Number of genes | Number of pairs |
| --- | --- | --- |
| DNA metabolism | 430 | 711 |
| RNA metabolism | 426 | 514 |
| amino acid and derivative metabolsim | 188 | 153 |
| carbon metabolism | 190 | 124 |
| cell budding | 77 | 44 |
| cell cycle | 508 | 855 |
| cell homeostasis | 106 | 57 |
| cell wall organization and biogenesies | 138 | 90 |
| cellular respiration | 86 | 19 |
| conjugation | 100 | 24 |
| cytokinesis | 96 | 44 |
| cyto skeleton organzition and biogenesis | 290 | 242 |
| electron transport | 21 | 4 |
| generation of precursor and energy | 222 | 245 |
| lipid metabolism | 201 | 73 |
| meiosis | 127 | 29 |
| membrane organization and biogenesis | 29 | 6 |
| morpogenesis | 140 | 82 |
| nuclear organzation and biogenesis | 60 | 2 |
| organalle organization and biogenesis | 944 | 3964 |
| protein biosysnthesis | 461 | 13117 |
| protein catabolism | 156 | 68 |
| protein modification | 390 | 326 |
| pseudohyphal growth | 48 | 9 |
| response of stress | 347 | 501 |
| ribosom biogenesis and assembling | 226 | 828 |
| signal transduction | 155 | 45 |
| sporulation | 94 | 36 |
| transciption | 465 | 535 |
| transport | 851 | 1907 |
| vesical mediated transport | 256 | 223 |
| vitamin metabolism | 72 | 14 |

Table S4. Distribution of function-similarity pairs inferred by the proposed method (with a p-value threshold of 2.7e-3) in each protein cellular function class of the database MIPS (only genes existing in the chosen Cho dataset are included in the table).

| Protein function | Number of genes | Number of pairs |
| --- | --- | --- |
| amino acid metabolism | 242 | 165 |
| nitrogen and sulfur metabolism | 91 | 26 |
| nucleotide metabolism | 225 | 197 |
| phosphate metabolism | 416 | 408 |
| C-compound and carbohydrate metabolism | 498 | 694 |
| lipid, fatty acid and isoprenoid metabolism | 265 | 119 |
| metabolism of vitamins, cofactors, and prosthetic groups | 161 | 80 |
| secondary metabolism | 70 | 8 |
| extracellular metabolism | 1 | 0 |
| glycolysis and gluconeogenesis | 54 | 41 |
| glyoxylate cycle | 9 | 0 |
| Entner-Doudoroff pathway | 0 | 0 |
| pentose-phosphate pathway | 23 | 1 |
| pyruvate dehydrogenase complex | 4 | 0 |
| anaplerotic reactions | 0 | 0 |
| tricarboxylic-acid pathway (citrate cycle, Krebs cycle, TCA cycle) | 31 | 3 |
| electron transport and membrane-associated energy conservation | 48 | 20 |
| respiration | 122 | 48 |
| fermentation | 48 | 15 |
| chemolithotrophie (e.g. sulfide, nitrogenous compounds) | 0 | 0 |
| metabolism of energy reserves (e.g. glycogen, trehalose) | 56 | 5 |
| oxidation of fatty acids | 6 | 0 |
| photosynthesis | 0 | 0 |
| energy conversion and regeneration | 35 | 3 |
| storage facilitating proteins | 0 | 0 |
| stored proteins | 0 | 0 |
| DNA processing | 500 | 867 |
| cell cycle | 638 | 1023 |
| RNA synthesis | 621 | 854 |
| RNA processing | 378 | 419 |
| RNA modification | 59 | 10 |
| ribosome biogenesis | 364 | 11314 |
| translation | 88 | 188 |
| translational control | 63 | 80 |
| aminoacyl-tRNA-synthetases | 37 | 10 |
| protein folding and stabilization | 90 | 51 |
| protein targeting, sorting and translocation | 280 | 245 |
| protein modification | 612 | 771 |
| assembly of protein complexes | 196 | 127 |
| protein degradation | 251 | 161 |
| protein binding | 372 | 322 |
| peptide binding | 3 | 0 |
| nucleic acid binding | 345 | 618 |
| polysaccharide binding | 0 | 0 |
| motor protein | 5 | 0 |
| structural protein | 52 | 10 |
| lipid binding | 17 | 0 |
| amino acid binding | 3 | 0 |
| sulfate binding | 0 | 0 |
| C-compound binding | 9 | 0 |
| metal binding | 28 | 4 |
| nucleotide binding | 221 | 107 |
| complex cofactor/cosubstrate binding | 55 | 9 |
| mechanism of regulation | 30 | 0 |
| target of regulation | 223 | 92 |
| transported compounds (substrates) | 567 | 1140 |
| transport facilitation | 184 | 113 |
| transport routes | 691 | 1316 |
| intracellular signalling | 193 | 86 |
| transmembrane signal transduction | 43 | 2 |
| stress response | 456 | 897 |
| disease, virulence and defense | 33 | 1 |
| detoxification | 111 | 50 |
| degradation of foreign (exogenous) compounds | 1 | 0 |
| ionic homeostasis | 171 | 108 |
| membrane excitability | 0 | 0 |
| cell motility | 0 | 0 |
| cell adhesion | 13 | 1 |
| cellular sensing and response | 284 | 157 |
| nutrients uptake and absorption (e.g. digestion) | 0 | 0 |
| osmoregulation and excretion | 2 | 0 |
| gas and metabolite distribution | 0 | 0 |
| systemic temperature regulation | 0 | 0 |
| systemic rhythm control | 0 | 0 |
| plant / fungal specific systemic sensing and response | 3 | 0 |
| animal specific systemic sensing and response | 3 | 0 |
| LTR retroelements (retroviral) | 0 | 0 |
| non-LTR retroelements | 0 | 0 |
| transposons | 0 | 0 |
| viral proteins | 0 | 0 |
| phage proteins | 0 | 0 |
| proteins necessary for the integration or inhibition of transposon movement | 8 | 0 |
| cell growth / morphogenesis | 245 | 192 |
| cell differentiation | 1 | 0 |
| dedifferentiation | 0 | 0 |
| cell death | 18 | 2 |
| cell aging | 28 | 1 |
| fungal/microorganismic development | 67 | 11 |
| plant development | 0 | 0 |
| animal development | 0 | 0 |
| cell wall | 218 | 140 |
| eukaryotic plasma membrane | 7 | 1 |
| cytoplasm | 1 | 0 |
| cytoskeleton | 261 | 184 |
| centrosome | 2 | 0 |
| cell junction | 0 | 0 |
| endoplasmic reticulum | 10 | 0 |
| Golgi | 7 | 0 |
| intracellular transport vesicles | 8 | 0 |
| nucleus | 158 | 94 |
| mitochondrion | 157 | 100 |
| peroxisome | 34 | 2 |
| endosome | 1 | 0 |
| vacuole or lysosome | 43 | 8 |
| plastid | 0 | 0 |
| extracellular / secretion proteins | 1 | 0 |
| periplasmatic space | 0 | 0 |
| bud / growth tip | 43 | 22 |
| prokaryotic cytoplasmic membrane | 5 | 0 |
| flagellum | 0 | 0 |
| pilus/fimbria | 0 | 0 |
| prokaryotic cell envelope structures | 0 | 0 |
| prokaryotic intracytoplasmic membrane | 0 | 0 |
| prokaryotic cell inclusions | 0 | 0 |
| prokaryotic nucleoid | 0 | 0 |
| fungal/microorganismic cell type differentiation | 453 | 591 |
| plant cell type differentiation | 0 | 0 |
| animal cell type differentiation | 0 | 0 |
| fungal/microorganismic tissue | 0 | 0 |
| plant tissue | 0 | 0 |
| animal tissue | 0 | 0 |
| fungal organ | 0 | 0 |
| plant organ | 0 | 0 |
| animal organ | 0 | 0 |
| cell wall | 0 | 0 |
| eukaryotic plasma membrane / membrane attached | 0 | 0 |
| cytoplasm | 0 | 0 |
| cytoskeleton | 0 | 0 |
| centrosome | 0 | 0 |
| cell junction | 0 | 0 |
| endoplasmic reticulum | 0 | 0 |
| Golgi | 0 | 0 |
| intracellular transport vesicles | 0 | 0 |
| nucleus | 0 | 0 |
| mitochondrion | 0 | 0 |
| peroxisome | 0 | 0 |
| endosome | 0 | 0 |
| vacuole or lysosome | 0 | 0 |
| plastid | 0 | 0 |
| extracellular / secretion proteins | 0 | 0 |
| periplasmatic space | 0 | 0 |
| bud / growth tip | 0 | 0 |
| prokaryotic cytoplasmic membrane | 0 | 0 |
| flagellum | 0 | 0 |
| pilus/fimbria | 0 | 0 |
| prokaryotic cell envelope component | 0 | 0 |
| prokaryotic intracytoplasmic membrane | 0 | 0 |
| prokaryotic cell inclusions | 0 | 0 |
| prokaryotic nucleoid | 0 | 0 |
| fungal / microorganismic cell type | 0 | 0 |
| plant cell type | 0 | 0 |
| animal cell type | 0 | 0 |
| fungal/microorganismic tissue | 0 | 0 |
| plant tissue | 0 | 0 |
| animal tissue | 0 | 0 |
| fungal organ | 0 | 0 |
| plant organ | 0 | 0 |
| animal organ | 0 | 0 |

Table S5. List of part of the regulatory interactions that cannot be significantly detected by the LC method and/or the PCC method but are detected by TC method with a p-value threshold of 1.3e-2.

| Regulator | Target gene | sc | cc | Relationship | Local clustering  score | Pearson  correlated coefficient | P-value  of  GWLA |
| --- | --- | --- | --- | --- | --- | --- | --- |
| YOL089C | YOL121C | 16 | 0.831122 | Negative | 9.9407458 | -0.55043 | 4.60E-04 |
| YER111C | YJL196C | 15 | 0.802232 | Positive | 11.538948 | 0.64534 | 1.10E-04 |
| YGL071W | YHL013C | 15 | 0.702164 | Negative | 8.9411401 | -0.30789 | 6.50E-04 |
| YGL071W | YKR026C | 15 | 0.696942 | Negative shift 1 | 6.8790766 | 0.40465 | 9.20E-06 |
| YER111C | YPL024W | 15 | 0.623852 | Positive | 7.5322609 | 0.41849 | 7.90E-06 |
| YKL043W | YCR098C | 15 | 0.551093 | Negative | 9.0289825 | -0.44967 | 2.30E-06 |
| YJL056C | YCR039C | 14 | 0.858194 | Negative | 11.268585 | -0.66286 | 7.80E-04 |
| YPL089C | YGR218W | 14 | 0.833044 | Negative shift 2 | 10.467281 | 0.12039 | 5.80E-04 |
| YDR423C | YLL060C | 14 | 0.816147 | Negative | 9.5961288 | -0.39143 | 6.10E-09 |
| YMR164C | YPR082C | 14 | 0.790786 | Negative shift 1 | 9.148612 | -0.069767 | 7.40E-04 |
| YMR164C | YKL008C | 14 | 0.786339 | Negative | 9.5726666 | -0.48135 | 4.80E-04 |
| YBR049C | YGL089C | 14 | 0.786231 | Negative | 10.321267 | -0.24474 | 7.40E-04 |
| YBL021C | YLL027W | 14 | 0.720557 | Positive | 9.248479 | 0.36266 | 2.70E-11 |
| YKL043W | YKL063C | 14 | 0.720033 | Positive | 9.7140216 | 0.55578 | 1.70E-04 |
| YMR043W | YKL058W | 14 | 0.708798 | Positive | 8.6601972 | -0.13215 | 7.40E-04 |
| YMR043W | YLR189C | 14 | 0.703429 | Negative shift 1 | 8.3665055 | -0.27692 | 1.90E-06 |
| YBL021C | YNL009W | 14 | 0.702171 | Negative | 10.230645 | -0.59078 | 1.70E-04 |
| YBR182C | YBL037W | 14 | 0.609683 | Positive | 9.1938386 | 0.45403 | 1.40E-04 |
| YER111C | YOR315W | 14 | 0.488102 | Negative shift 1 | 11.870964 | -0.55708 | 1.20E-05 |
| YKL112W | YDL012C | 14 | 0.471585 | Negative shift 1 | 10.066975 | -0.21901 | 3.20E-04 |
| YKL043W | YMR020W | 14 | 0.436344 | Negative shift 1 | 11.368332 | -0.48792 | 3.90E-07 |
| YER111C | YOL019W | 13 | 0.938458 | Positive | 10.94639 | 0.54274 | 8.60E-04 |
| YNL027W | YGL038C | 12 | 0.973186 | Negative | 10.513213 | -0.5013 | 8.60E-04 |
| YPR065W | YML056C | 12 | 0.942039 | Positive shift 2 | 8.3247375 | -0.39056 | 8.50E-04 |
| YDL170W | YEL023C | 12 | 0.933047 | Positive | 9.9124424 | 0.50061 | 5.80E-04 |
| YMR043W | YML053C | 12 | 0.925863 | Positive | 10.047183 | 0.58961 | 1.20E-06 |
| YPL049C | YGR014W | 12 | 0.922706 | Negative shift 1 | 8.3105452 | 0.26673 | 3.60E-04 |
| YLR131C | YML007W | 12 | 0.919555 | Positive shift 1 | 10.983208 | 0.23229 | 5.30E-04 |
| YLR131C | YJR147W | 11 | 0.950579 | Negative shift 2 | 7.6481637 | 0.26497 | 2.00E-05 |
| YPR104C | YBR126C | 10 | 0.98278 | Negative shift 1 | 10.321439 | -0.60714 | 4.20E-04 |
| YMR042W | YDR434W | 10 | 0.978256 | Negative | 8.9156096 | -0.52445 | 9.20E-04 |
| YKL112W | YJL111W | 10 | 0.967586 | Negative | 7.2011332 | -0.20003 | 2.30E-04 |
| YOR028C | YOL116W | 10 | 0.955914 | Negative shift 1 | 8.1702864 | -0.32806 | 9.60E-04 |
| YDL056W | YER111C | 14 | 0.742521 | Negative | 13.868252 | -0.78333 | 3.60E-05 |
| YNL068C | YPL117C | 13 | 0.927805 | Negative | 12.289813 | -0.72293 | 2.30E-05 |
| YDR207C | YDR285W | 15 | 0.783501 | Negative shift 1 | 8.769669 | -0.15794 |  |
| YML027W | YKL091C | 15 | 0.718253 | Negative | 10.000145 | -0.58824 |  |
| YGL035C | YBR050C | 15 | 0.627898 | Positive | 8.3484768 | 0.33049 |  |
| YDR501W | YDL112W | 15 | 0.471435 | Positive | 8.2856154 | 0.29687 |  |
| YDR207C | YGR157W | 14 | 0.782776 | Negative | 10.267111 | -0.58183 |  |
| YGL096W | YAL058W | 14 | 0.78003 | Positive | 10.549761 | 0.61108 |  |
| YGL013C | YDR406W | 14 | 0.777687 | Positive shift 1 | 8.9293663 | 0.26907 |  |
| YBR049C | YGL026C | 14 | 0.758888 | Negative | 11.518361 | -0.13874 |  |
| YML027W | YFR011C | 14 | 0.723312 | Negative | 9.5680719 | -0.45911 |  |
| YER040W | YJR152W | 14 | 0.658305 | Positive shift 1 | 8.2257016 | -0.062523 |  |
| YIL122W | YBR161W | 14 | 0.601617 | Negative | 9.0160466 | -0.41813 |  |
| YML027W | YML006C | 14 | 0.597894 | Positive | 7.8484256 | 0.22884 |  |
| YOR344C | YBR195C | 14 | 0.584624 | Negative shift 1 | 8.4034377 | 0.12185 |  |
| YIL122W | YGR015C | 14 | 0.572323 | Negative | 10.6481 | -0.59923 |  |
| YOR372C | YGL226W | 14 | 0.571277 | Positive shift 1 | 9.990383 | 0.29061 |  |
| YER040W | YFL021W | 14 | 0.55537 | Positive | 10.969898 | 0.64529 |  |
| YOR344C | YAR073W | 14 | 0.545081 | Negative shift 1 | 5.5138459 | 0.22707 |  |
| YBL021C | YOR375C | 14 | 0.480163 | Negative shift 1 | 9.1763075 | 0.13332 |  |
| YOR372C | YCL028W | 14 | 0.480023 | Negative | 10.327765 | -0.55964 |  |
| YOR372C | YDR471W | 14 | 0.404909 | Negative | 7.1805473 | -0.28519 |  |
| YGL096W | YDL057W | 13 | 0.918778 | Positive shift 1 | 10.751163 | 0.61501 |  |
| YLR183C | YNR039C | 12 | 0.948483 | Negative | 10.721158 | -0.59372 |  |
| YDL106C | YGL234W | 11 | 0.960577 | Negative shift 1 | 11.497533 | -0.3548 |  |
| YDR451C | YJL115W | 11 | 0.956276 | Positive shift 2 | 11.799786 | -0.11328 |  |
| YDR501W | YDR328C | 11 | 0.951962 | Positive shift 1 | 8.6092633 | -0.11041 |  |
| YDR123C | YNR016C | 11 | 0.945824 | Negative | 8.6111669 | 0.012985 |  |
| YGL096W | YOL077C | 11 | 0.945578 | Negative | 8.2264142 | -0.41821 |  |
| YKL038W | YGL062W | 10 | 0.954995 | Positive shift 1 | 10.797557 | -0.25253 |  |
| YLR183C | YCL027W | 10 | 0.952344 | Negative shift 1 | 9.0499788 | 0.19728 |  |
| YML007W | YHR008C | 10 | 0.950265 | Positive shift 1 | 9.1433079 | 0.28109 |  |
| YDR451C | YGL114W | 15 | 0.594549 | Negative shift 1 | 12.300286 | -0.29749 |  |
| YDR207C | YAL054C | 14 | 0.669631 | Negative | 12.173642 | -0.70959 |  |
| YDR123C | YBR093C | 11 | 0.958086 | Negative | 13.906861 | -0.81805 |  |
| YLR183C | YPR139C | 11 | 0.938342 | Negative shift 2 | 12.149101 | 0.34538 |  |

Note: There are some blank in the column p-value of GWLA because the corresponding interactions are found by the other methods and from regulatory interaction collection dataset.

Table S6. Normalized expression value at each time point in the gene RCS1 and GCN3 in Fig.4A (in the text).

| Time point | 1 | 2 | 3 | 4 | 5 | 6 | 7 | 8 | 9 | 10 | 11 | 12 | 13 | 14 | 15 | 16 | 17 |
| --- | --- | --- | --- | --- | --- | --- | --- | --- | --- | --- | --- | --- | --- | --- | --- | --- | --- |
| RCS1 | -0.37976 | -1.4387 | 0.4742 | -1.1312 | -0.31144 | 0.54252 | -1.3362 | -0.5164 | -0.7555 | 0.26925 | -0.68719 | -0.27729 | 1.9089 | 1.294 | -0.48224 | 1.1915 | 1.6356 |
| GCN3 | -1.101 | -1.5485 | 0.93343 | 0.39092 | 0.52655 | -1.5621 | -1.9825 | 1.0419 | 0.17392 | 0.54011 | -0.62628 | 0.59436 | -0.12446 | -0.24652 | 0.41805 | 1.3403 | 1.2318 |

Pearson correlation coefficient (PCC) is 0.40465 according to $\frac{1}{17}\sum_{i=1}^{17} X_{i}Y_{i}$ because here the expression levels are normalized to $X{}_{i}$,$Y_{i}$ in the “z score” fashion (X represents RCS1).

The results of local clustering (LC) according to the algorithm (Qian, et al., [8]) are

|  | max_score | startx | starty | len | relationship |
| --- | --- | --- | --- | --- | --- |
|  | 6.8791 | 17 | 17 | 17 | 1 |

The max_score is the final score of LC; 1 means positive relationship (details in Qian, et al., [8]).

The maximal local alignment of expression change trend between the two genes is 15 and cc is 0.70 according to the algorithm of TC in the supplementary materials and text.
